# Temperature Dependence of Carrier Extraction Processes in GaSb/AlGaAs Quantum Nanostructure Intermediate-Band Solar Cells

**DOI:** 10.3390/nano11020344

**Published:** 2021-01-29

**Authors:** Yasushi Shoji, Ryo Tamaki, Yoshitaka Okada

**Affiliations:** 1 Global Zero Emission Research Center, National Institute of Advanced Industrial Science and Technology (AIST), Tsukuba, Ibaraki 305-8568, Japan; 2 Research Center for Advanced Science and Technology (RCAST), The University of Tokyo, Meguro-ku, Tokyo 153-8904, Japan; tamaki@mbe.rcast.u-tokyo.ac.jp (R.T.); okada@mbe.rcast.u-tokyo.ac.jp (Y.O.)

**Keywords:** intermediate-band solar cell, quantum dot, molecular-beam epitaxy

## Abstract

From the viewpoint of band engineering, the use of GaSb quantum nanostructures is expected to lead to highly efficient intermediate-band solar cells (IBSCs). In IBSCs, current generation via two-step optical excitations through the intermediate band is the key to the operating principle. This mechanism requires the formation of a strong quantum confinement structure. Therefore, we focused on the material system with GaSb quantum nanostructures embedded in AlGaAs layers. However, studies involving crystal growth of GaSb quantum nanostructures on AlGaAs layers have rarely been reported. In our work, we fabricated GaSb quantum dots (QDs) and quantum rings (QRs) on AlGaAs layers via molecular-beam epitaxy. Using the Stranski–Krastanov growth mode, we demonstrated that lens-shaped GaSb QDs can be fabricated on AlGaAs layers. In addition, atomic force microscopy measurements revealed that GaSb QDs could be changed to QRs under irradiation with an As molecular beam even when they were deposited onto AlGaAs layers. We also investigated the suitability of GaSb/AlGaAs QDSCs and QRSCs for use in IBSCs by evaluating the temperature characteristics of their external quantum efficiency. For the GaSb/AlGaAs material system, the QDSC was found to have slightly better two-step optical excitation temperature characteristics than the QRSC.

## 1. Introduction

With increasing interest in a low-carbon society, the development of solar cells (SCs) with high power generation efficiency has become urgent. As novel photovoltaic devices for realizing a high conversion efficiency that exceeds the limit of conventional SCs, intermediate-band solar cells (IBSCs) have recently become the focus of attention [1,2,3,4]. The operating principle of IBSCs involves optical transition via sub-bandgap intermediate-band (IB), which can absorb photons with an energy that is less than the difference between the conduction band (CB) and valence band (VB) of the host material, thereby increasing the photocurrent. The IB formed between CB and VB has also been used to obtain interfacial conductivity in wide-gap material devices, in which IB functions as a pathway for charge transfer [5]. However, in IBSCs, two-step optical absorption through IB is key to the operation. The theoretical conversion efficiency of standard single-junction SCs without IB depends on the bandgap of the absorber. For SCs with small bandgaps, a high output current can be obtained because there are few photons transmitted through them, whereas the output voltage becomes small because it is determined by the quasi-Fermi level splitting of the absorber. The opposite occurs if the band gap of the absorber is large. In ideal IBSCs, the quasi-Fermi levels of CB, VB, and IB are independent of each other. This is achieved by the carrier transitions of VB–IB and IB–CB, which occur by optical excitations rather than thermal excitations. As a result, ideal IBSCs can obtain a current gain without reducing the host material voltage owing to the absorption of photons with energy lower than the bandgap of the host material using IB. The use of a quantum dot (QD) superlattice has been intensively investigated as a method for forming IBs [6,7,8,9,10]. Although photocurrent production by two-step optical excitation via QD states in QDSCs has been reported, the contribution of this two-step excitation to the conversion efficiency is still small [11,12,13].

Ideally, in the process of extracting carriers generated in the IB, the processes involving thermal excitation and electric-field assistance should be suppressed as much as possible and the processes involving optical excitation should be dominant. Therefore, the following structure is preferable for QD-IBSCs: First, to prevent electric-field-induced carrier escape from the IB, the application of the strong built-in electric field of the *p*–*n* junction to the IB should be avoided. We previously reported that the electric-field strength applied to the IB can be controlled by a field-damping layer with a low carrier concentration and that the IB can thus be formed nearly flat [14,15].

Second, a higher band offset energy between the QDs and barrier layers is needed to suppress the thermal excitation of carriers in the IB. This is important for obtaining the high-temperature operation of two-step optical excitation via IB. Since solar cells are usually used for terrestrial applications at room temperature or higher, the thermal escape rate of carriers absorbed within IB increases due to the high thermal energy at such temperatures. As mentioned above, the main principle of IBSC is to obtain the current gain without any voltage loss using optical transitions through IB, which can be achieved by separating the quasi-Fermi levels of CB, VB, and IB. A high thermal escape rate case means that the quasi-Fermi levels are not well separated, leading to a decrease in the output voltage [16]. InAs quantum dots embedded by GaAs layers are among the most typical QDs in III-V materials, and their growth technology is more mature than that of other materials. Therefore, IBSCs have been vigorously studied in InAs/GaAs QDs. However, effective current production due to two-step absorption has not been achieved at higher temperatures because of the small band offset between QD layers and the host material of GaAs. Ramiro et al. demonstrated the suppression of the thermal escape of carriers generated in QDs using wide-gap AlGaAs as a host material [17]. They reported that the higher band offset between InAs and AlGaAs suppresses the carrier thermal escape up to a temperature of 220 K. In addition, our previous work demonstrated the importance of increasing the band offset to obtain two-step optical absorption at higher temperatures [18], where the threshold temperature of the two-step optical absorption was defined as the temperature of the photocurrent intensity with 1% of the signal obtained at 9 K. According to this evaluation, changing the host material of InAs QDs from GaAs to wider-gap AlGaAs improves the threshold temperature of the two-step absorption from 82 to 139 K. Furthermore, a threshold temperature of 220 K was obtained in InGaAs/AlGaAs QDSCs, which were tuned for stronger confinement. If not limited to III-V materials, two-step optical absorption at room temperature has recently been obtained in IBSCs with lead sulfide QDs and wide-gap lead halide perovskites [19]. However, perovskite materials have some issues in terms of long-term stability. Among III-V semiconductors, wide-gap materials include AlGaAs, InGaP, and GaAsP. When phosphide materials are used as barrier layers for InAs QDs, the interface quality is often a problem due to the intermixing of As and P atoms. Therefore, a method for introducing a thin interlayer to improve the interface quality between QDs and barrier layers has been proposed [20,21,22]. 

Third, the carriers in the IB should have a sufficiently long lifetime to avoid rapid recombination. Therefore, a type-II quantum structure, in which electrons and holes are spatially separated, is preferable [23,24,25,26,27]. GaSb/AlGaAs type-II QDs are expected to exhibit the aforementioned preferred features. In this system, the holes are confined in GaSb QDs surrounded by AlGaAs barrier layers [28]. More interestingly, GaSb QDs can undergo a change in shape to quantum rings (QRs) when irradiated with an As molecular beam because of the desorption of Sb from the QDs, which occurs via As/Sb exchange [29,30]. We previously reported that GaSb QRs have less local lattice strain than GaSb QDs, resulting in better overall crystal quality of the multi stacked structure in a GaSb/GaAs system [31]. In the present paper, we first investigate the growth and structural properties of GaSb quantum nanostructures on an AlGaAs layer and then discuss whether the AlGaAs barrier layer is effective in improving two-step optical excitation in the GaSb QDs. In addition, the difference in the temperature dependence of the two-step optical excitation between GaSb/AlGaAs QDs and QRs is investigated.

## 2. Experimental Procedure

All samples were grown on GaAs (001) substrates via solid-source molecular-beam epitaxy. We first attempted to grow GaSb quantum nanostructures on an Al_0.2_Ga_0.8_As layer. After the semi-insulating GaAs substrate was thermally cleaned at 590 °C, a 250-nm-thick buffer layer was grown at 580 °C. Then, 2.5 monolayers (MLs) GaSb was grown on a 200-nm-thick Al_0.2_Ga_0.8_As layer at 480 °C. The Sb beam pressure was 6 × 10^−5^ Pa. The growth rate of GaSb was 0.6 ML/s. We confirmed that self-organized GaSb QDs could be formed via the Stranski–Krastanov growth mode under these growth conditions [32,33]. Moreover, samples based on the aforementioned structure were prepared by irradiation with an As molecular beam with a beam pressure 1 × 10^−4^ Pa for 0–30 s after GaSb growth to investigate the shape change of the quantum nanostructures to QRs. Similarly, samples with GaSb nanostructures grown at a substrate temperature of 460 °C were also prepared.

**Figure 1:**
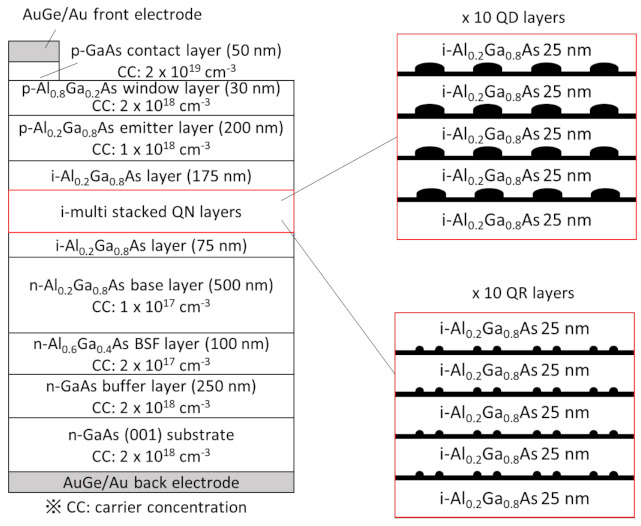
Schematic of the cell structures fabricated in the present study. The device structures consist of *p–i–n* layers. Multilayers of GaSb/AlGaAs quantum nanostructures were inserted into the *i*-layer.

We also fabricated *p*–*i*–*n*-structured QD- and QR-IBSCs using AlGaAs as a host material. Figure 1 shows the device design of the IBSCs. After the *n*-GaAs substrate was thermally cleaned at 590 °C, a 250-nm-thick *n*-GaAs buffer layer, a 100-nm-thick *n*-Al_0.6_Ga_0.4_As back-surface field (BSF) layer, and a 500-nm-thick *n*-Al_0.2_Ga_0.8_As base layer were grown at 580 °C. The Al_0.6_Ga_0.4_As BSF layer was introduced to reduce the recombination of minority carriers near the backside. Then, 10 layers of GaSb/Al_0.2_Ga_0.8_As QDs or QRs were subsequently introduced into the *i*-layer. The multi stacked structures were grown at 460 °C. The 150-nm-thick *p*-Al_0.2_Ga_0.8_As emitter layer, 30-nm-thick *p*-Al_0.8_Ga_0.2_As window layer, and a 50-nm-thick *p*-GaAs contact layer were then grown. In all of the layers, Be and Si were used as *p*-type and *n*-type dopants, respectively. Ga and Al were evaporated using hot-lip-type dual-filament and single-filament effusion cells, respectively. As and Sb were supplied by cracker effusion cells that enabled valve control of the flux.

**Figure 2:**
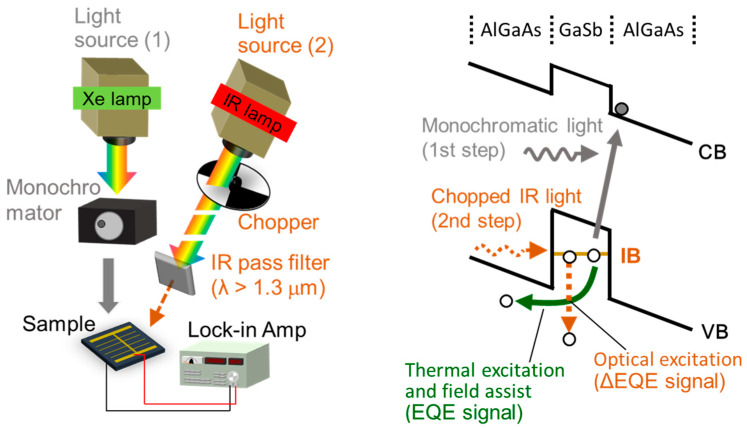
Measurement setup for characterization of photocurrent production due to each carrier extraction process. One of the light sources provides continuous-wave monochromatic photons that generate electron–hole pairs in the VB–CB and IB–CB (Light source (1)). Chopped IR lamp with a long-pass filter positioned at the exit passes through a set of filters that collectively allow only photons of *λ* > 1300 nm to be transmitted (Light source (2)). The illumination of low-energy photons from this IR source can then only pump the electrons from the IB to the VB.

After the growth, AuGe/Au was used as *n*-type and *p*-type Ohmic electrodes. The mesa-type structures were formed by chemical wet-etching using a solution with a H_3_PO_4_:H_2_O_2:_H_2_O ratio of 4:1:20. Finally, a *p*-type GaAs contact layer was etched by a citric-acid-based solution in areas where the electrodes were not deposited.The structural properties of the quantum nanostructures were analyzed by atomic force microscopy (AFM) (Seiko Instruments Inc., E-Sweep, Chiba, Japan). The device performance was characterized using current–voltage curves measured under air mass 1.5 global (AM 1.5G) solar spectrum illumination. The optical characteristics were evaluated using a unique external quantum efficiency (EQE) measurement system (Figure 2). One of the light sources provided continuous-wave monochromatic photons that generated electron–hole pairs in the host material and quantum nanostructures. The EQE spectra were acquired by changing the wavelength of this monochromatic light. The other light source was a chopped infrared (IR) lamp with a long-pass filter that allowed only photons with a wavelength *λ* longer than 1.3 μm to pass through. The illumination of low-energy photons from this IR source provided the broadband excitation and pumped holes from the IB formed by quantum nanostructures to the VB. The photocurrent production due to two-step optical excitation via the IB was estimated by measuring the difference between the EQE with and without this IR illumination. The difference in EQE (ΔEQE) under IR illumination was detected using a lock-in amplifier synchronized (NF Corp. LI5655, Yokohama, Japan) with an optical chopper (Thorlabs Inc. MC2000, Newton, NJ, USA) set to 85 Hz. The measurement was performed in a cryostat.

## 3. Results and Discussion

### 3.1. Structural Properties of GaSb Quantum Nanostructures Grown on AlGaAs Layers

**Figure 3:**
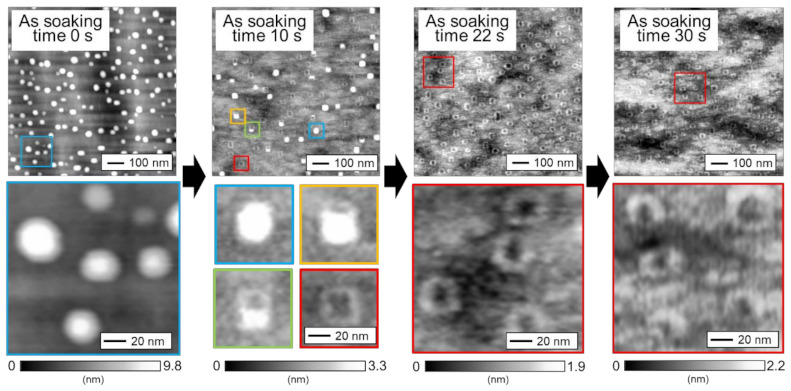
Plane view of AFM images for GaSb quantum nanostructures grown on an AlGaAs layer at 480 °C. The As-soaking time after the deposition of GaSb was varied.

We first conducted AFM measurements to confirm the structure of the GaSb QDs and QRs grown on Al_0.2_Ga_0.8_As layers. Figure 3 shows an AFM image for a single layer of GaSb quantum nanostructures grown on an Al_0.2_Ga_0.8_As layer at 480 °C. These samples were fabricated at different As-soaking times after growth of the GaSb. Lens-shaped QD structures were observed for the sample without As-soaking, whereas a mixture of QDs, QRs, and their intermediate structures was observed for the sample with an As-soaking time of 10 s. For the samples with an As-soaking time of 22 and 30 s after growth of the GaSb QDs, all of the QDs were transformed into QRs via the As/Sb exchange reaction. This result suggests that the shape of the GaSb quantum nanostructures can be changed by the As-soaking, even when the nanostructures are on an Al_0.2_Ga_0.8_As layer. The exchange of the As and Sb atoms occurred because the bond between Ga and Sb atoms was much weaker than the bond between Ga and As atoms [34]. Lin et al. reported the transformation mechanism of GaSb QDs to QRs [30]. In their study, Sb with background As soaking was performed after the growth of GaSb QDs. During the soaking, As atoms were aggregated on the summit of the GaSb QDs, and such As-rich region induced lattice mismatch strain to the underlying GaSb QD. Hence, the Sb atoms were diffused away to relax the strain energy. Due to the larger strain on the summits of the QDs, most of the Sb atoms were repelled from the QDs. As a result, a ring structure was formed. The detailed structure of GaSb QR was reported by Timm et al., who analyzed the composition of QR and observed a clear central opening structure through scanning tunneling microscopy (STM) [29].

**Figure 4:**
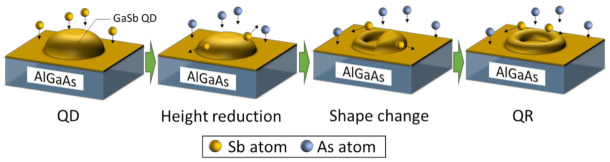
A model for the transformation of GaSb QDs into QRs, as inferred from this study.

In the present work, the transition from QDs to QRs appears to proceed as shown in Figure 4 because quantum nanostructures of various shapes are mixed together in the samples with an As-soaking time of 10 s. Immediately after the supply of As after the growth of GaSb QDs, the QD height decreases. The transition then progresses to a shape change as the height falls below a certain threshold. Subsequently, according to the transformation mechanism described above, the structure stabilizes in the ring shape when sufficient As atoms have been supplied. In the sample without As-soaking, QDs of various heights were formed, with an average height of 5.70 nm (in-plane density: 1.7 × 10^10^ cm^−2^). Therefore, the extent of the shape change differed for each QD size. In the case of an As-soaking time of 10 s, QDs with a large height decreased in height but did not undergo a change in shape. However, the QDs with a small height decreased sufficiently in height for their shape to change even with a short As irradiation time of 10 s, leading to the formation of a ring shape. For the sample with an As-soaking time of 22 s, all of the QDs in the plane transformed into QRs because sufficient As was supplied to induce even the larger QDs to transform into QRs. The AFM image of the sample with the As-soaking time of 10 s shows the change process to QR. As shown in the shape change process of Figure 4, unlike the described trends in previous reports, a ring shape was formed from the side of the QDs rather than from the center, considerably due to the fact that the lattices of the GaSb crystal were anisotropically strained. Actually, the QDs grown in the present study did not have an isotropic shape, and the shape change by the As-soaking proceeded from the longitudinal direction. However, to clarify the main cause and prove the hypothesis, a further detailed structural analysis, such as STM, would be necessary. 

**Figure 5:**
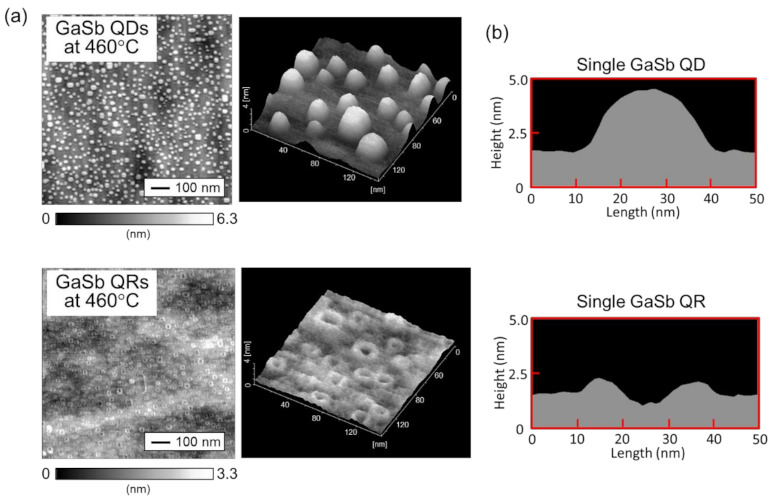
(**a**) Plane and bird’s eye views of AFM images of GaSb QDs and QRs grown on an AlGaAs layer at 460 °C. (**b**) Line profiles of a single GaSb QD and QR.

Because growing multi stacked structures with large QDs for IBSC is difficult, we next attempted to grow the small QDs with a high in-plane density by decreasing the growth temperature. In addition, we verified that QR structures can also be fabricated at high density. Figure 5a shows GaSb quantum nanostructures grown on an AlGaAs layer at 460 °C. Table 1 shows the mean diameter, height, and density of QDs and QRs, as determined from AFM measurements. Herein, for the QRs, the outer diameters were measured. For the sample grown at 460 °C, small QDs were confirmed to form at a density greater than in the sample grown at 480 °C. This greater density is attributed to the lower growth temperature shortening the surface migration length of Ga atoms and facilitating the formation of small QDs. Moreover, the density of GaSb QRs also increased with decreasing growth temperature. Figure 5b shows the line profiles of a single QD and QR. The diameter of the QR structure was approximately the same as that of the QD structure, whereas the height of the QR structure was substantially smaller than that of the QD structure.

### 3.2. Characterization of the Device Performances and Carrier Extraction Processes for GaSb/AlGaAs QDSCs and QRSCs

**Figure 6:**
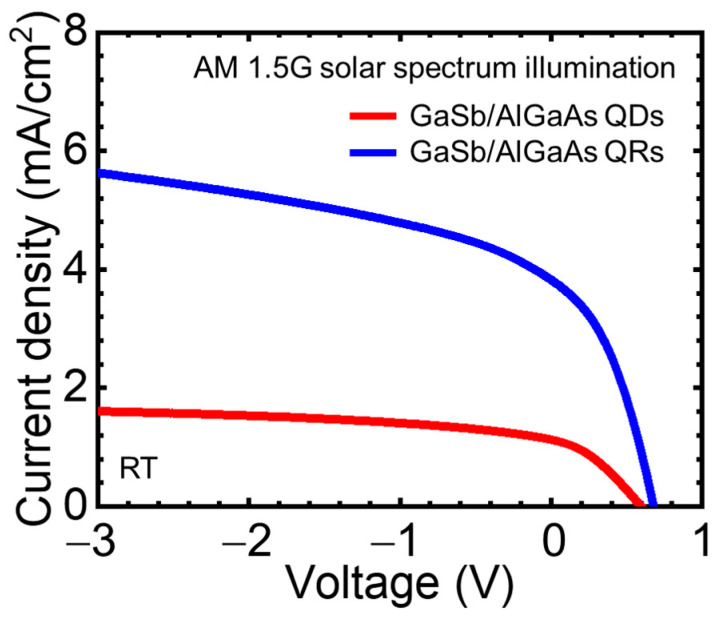
Current–voltage curves for the GaSb/AlGaAs QDSCs and QRSCs measured under AM 1.5 G solar spectrum illumination.

Figure 6 shows the current–voltage curves of the GaSb/AlGaAs QDSCs and QRSCs, which were measured under AM 1.5 G solar spectrum illumination. The measurements were carried out at room temperature. The short-circuit current density (*J*_SC_), open-circuit voltage (*V*_OC_), and fill factor (FF) of both cells are summarized in Table 2. The trend due to the difference in the quantum nanostructure is the same as that of the GaSb/GaAs system. Owing to the difference in the crystal quality, QRSCs were superior in terms of the device performance compared with QDSCs. Furthermore, both SCs with the AlGaAs host material showed higher V_OC_ compared with the SCs with the GaAs host material [31]. However, in the operation of IBSCs, obtaining a two-step optical transition via IB at higher temperatures is important. Hence, we investigated the temperature dependence of the carrier extraction processes for each SC by measuring EQE and ΔEQE.

**Figure 7:**
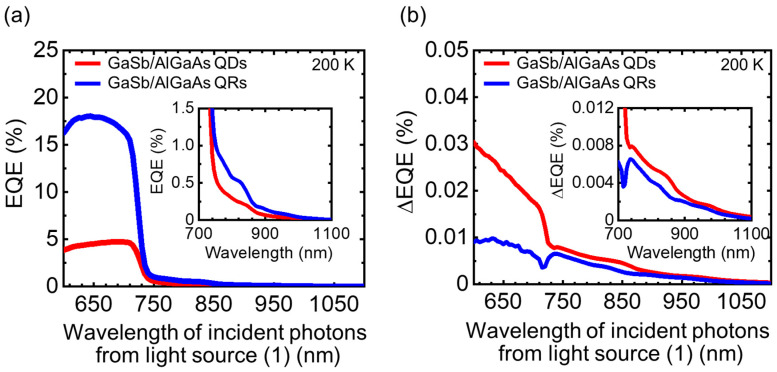
(**a**) EQE and (**b**) ΔEQE spectra for 10-layer stacked GaSb/AlGaAs QDSC and QRSC. The measurement temperature was 200 K. The insets show zoom-in of the photoabsorption region for GaSb quantum nanostructures.

Figure 7 shows (Figure 7a) EQE and (Figure 7b) ΔEQE spectra for GaSb/AlGaAs QDSCs and QRSCs, as obtained at 200 K. For both samples, the EQE responses in the wavelength region from 600 to 740 nm represent the photocurrent production due to the VB–CB optical excitation in the AlGaAs host material. In addition, the EQE signals in the wavelength range longer than 740 nm represent currents produced by the escape of photogenerated carriers in GaSb quantum nanostructures (IB) due to thermal excitation or electric-field assistance. Compared with the QDSC, the QRSC exhibits a higher EQE response associated with the AlGaAs host material. This difference is attributed to the volume of the QR structure being smaller than that of the QD structure, which results in less residual strain during stacking and enables the growth of an AlGaAs layer with fewer crystal defects. In the EQE signals with *λ* > 740 nm, the absorption volume of quantum nanostructures and the carrier collection efficiency of each extraction process compete. The ΔEQE signals in the 600 < *λ* < 740 nm range are associated with the IR-light-induced re-excitation of the holes that relaxed to the IB after being generated in the AlGaAs host material. The ΔEQE signals with *λ* > 740 nm are associated with photocurrents produced by the two-step optical excitation via IB. In the wavelength region of absorption by quantum nanostructures, samples with a low EQE and high ΔEQE are better suited for IBSC operation.

Furthermore, because SCs are, in principle, used at room temperature or higher, current generation due to two-step optical excitation via IB must occur at higher temperatures. Thus, we evaluated the temperature dependence of each photocurrent production process associated with the quantum nanostructures as below. The photocurrent production due to thermal excitation (*J*_TE_) and that due to field-assisted escape (*J*_FA_) from the IB to the VB were calculated by

(1) $$J_{\mathrm{TE}}+J_{\mathrm{FA}}=q\int_{\lambda_{CB-VB\_edge}}^{\lambda_{CB-IB\_edge}}\Phi \left(\lambda \right)\cdot \mathrm{EQE}\left(\lambda \right)\cdot d\lambda ,$$

where *λ_CB-VB_edge_* is the photoabsorption edge of the CB–VB determined by the EQE spectrum. *λ_CB–IB_edge_* is the photoabsorption edge of the CB–IB transition and the longest wavelength at which the EQE signal is observed. *q* is the elementary charge and *ϕ*(*λ*) is the photon flux. The photocurrent produced by optical excitation (*J*_OE_) from the IB to the VB was calculated by

(2) $$J_{\mathrm{OE}}=q\int_{\lambda_{CB-VB\_edge}}^{\lambda_{CB-IB\_edge}}\Phi \left(\lambda \right)\cdot ∆\mathrm{EQE}\left(\lambda \right)\cdot d\lambda ,$$

**Figure 8:**
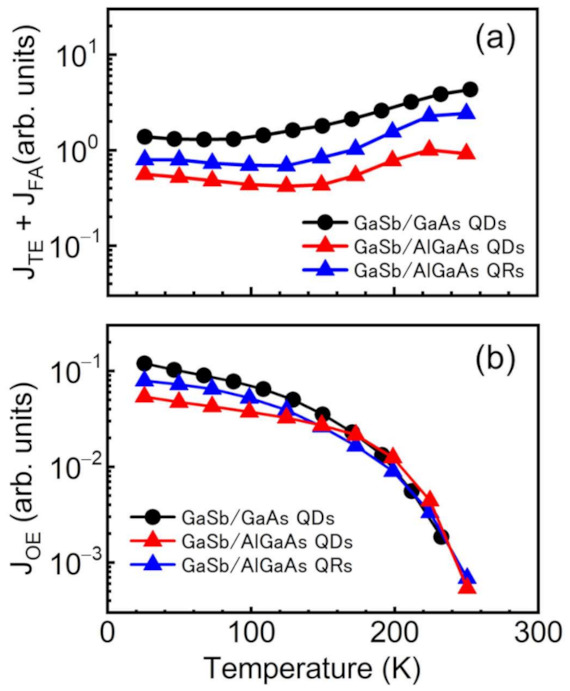
(**a**) Temperature dependence of the *J*_TE_ and *J*_FA_ for a 10-layer stacked GaSb/GaAs QDSC, GaSb/AlGaAs QDSC, and GaSb/AlGaAs QRSC. (**b**) Temperature dependence of the *J*_OE_ for a 10-layer stacked GaSb/GaAs QDSC, GaSb/AlGaAs QDSC, and GaSb/AlGaAs QRSC.

Figure 8a shows the temperature dependence of the sum of *J*_TE_ and *J*_FA_ for the GaSb/AlGaAs QDSC and QRSC. In addition, Figure 8a includes data for GaSb/GaAs QDs to compare the effect of the AlGaAs as the host material. The sum of *J*_TE_ and *J*_FA_ increased with increasing temperature for all of the samples. In the extremely low temperature region, the *J*_FA_ is dominant in current production because the thermal excitation is less likely to occur as a result of the low thermal energy. When sufficient thermal energy is generated to enable excitation of the carrier from the IB to the VB, the *J*_TE_ component is obtained and increases with increasing temperature. For the GaAs/GaSb QDSC, the sum of *J*_TE_ and *J*_FA_ increases when the temperature exceeds 100 K, indicating the initiation of thermal excitation. The onset temperature of thermal excitation for the samples with the AlGaAs host material shifts to higher temperatures compared with that for the samples with the GaAs host material. This result indicates that the use of a wide-bandgap material for the barrier layer of quantum nanostructures reduces undesirable carrier escape from the IB to the VB by thermal excitation. Figure 8b shows the temperature dependence of the *J*_OE_ for the GaSb/GaAs QDSC and the GaSb/AlGaAs QDSC and QRSC. For all of the samples, the *J*_OE_ decreases with increasing temperature because the component of the thermal extraction amount increases with increasing temperature. Herein, the reduction curve of *J*_OE_ includes the influence of decreased carrier collection efficiency by an increase in the nonradiative recombination rate because of the increase in temperature. The decrease in *J*_OE_ with increasing temperature for the GaSb/AlGaAs QDSC was slower than those for the other samples, indicating the feasibility of IBSC operation at higher temperatures. 

To clarify the suitability for IBSC operation, we evaluated the proportion of the optical excitation component (*P*_OE_) in the photocurrent production related to the IB–VB transitions from the following equation:

(3) $$P_{\mathrm{OE}}=\frac{J_{\mathrm{OE}}}{\left(J_{\mathrm{OE}}+J_{\mathrm{TE}}+J_{\mathrm{FA}}\right)}$$

**Figure 9:**
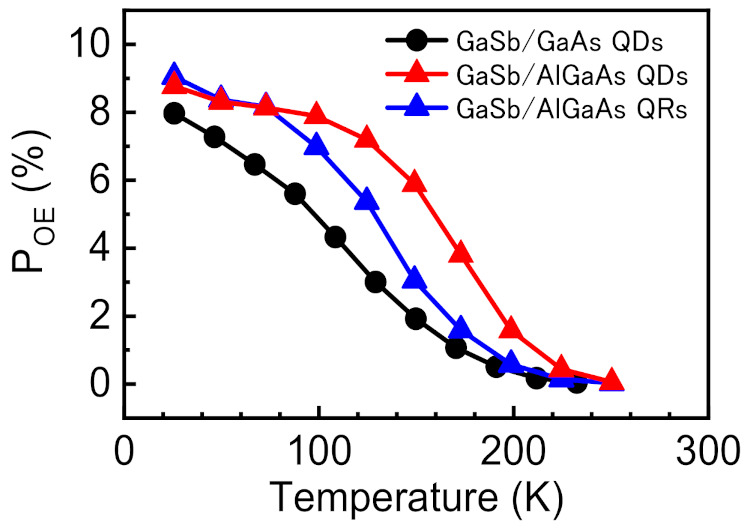
Temperature dependence of the *P*_OE_ for a 10-layer stacked GaSb/GaAs QDSC, GaSb/AlGaAs QDSC, and GaSb/AlGaAs QRSC.

A higher value of *P*_OE_ means a more suitable structure for IBSC. Figure 9 shows the temperature dependence of *P*_OE_ for the GaSb/GaAs QDSC and the GaSb/AlGaAs QDSC and QRSC.At lower temperatures, the *P*_OE_ is higher for samples with the AlGaAs host material than for the GaSb/GaAs QDSC. The *P*_OE_ curve of the GaSb/GaAs QDSC decreases substantially with increasing temperature; when the temperature is increased to 150 K, the value decreases to one-fourth of that at 26 K. By contrast, the GaSb/AlGaAs QDSC maintained a *P*_OE_ value of 6% even at 150 K. A higher energy was required for carrier escape by thermal excitation in the IB–VB transition for samples with the AlGaAs host material. Thus, carrier extraction by optical excitation occurred even at high temperatures. Moreover, the temperature characteristic of the *P*_OE_ for the GaSb/AlGaAs QDSC was superior to that of the *P*_OE_ for the GaSb/AlGaAs QRSC. This difference is attributed to the confinement strength of the quantum nanostructure. As shown in Table 1, the height of the QR structure is only 0.95 nm, whereas the height of the QD structure is 2.56 nm. Thus, compared with the IB in the QD structure, that in the QR structure is formed at a higher energy position within the potential. The results demonstrate that the QDSC prepared with wide-bandgap host-layer materials are suitable for IBSC operation. However, GaSb QDs tend to form defects in the host material because of the growth of multilayer structures; therefore, further research into growth conditions and structures (e.g., introduction of an interlayer between GaSb and AlGaAs [20,21,22]) that result in high-quality QDSCs is needed.

## 4. Conclusions

We studied 10-layer stacked GaSb/AlGaAs QDSCs and QRSCs for use in IBSCs. GaSb QDs and QRs were grown on an AlGaAs layer. We demonstrated that GaSb QDs and QRs can be formed on AlGaAs layers by Stranski–Krastanov growth and As-soaking techniques. Further, we evaluated the suitability of the GaAs/GaAs QDSC and the GaSb/AlGaAs QDSC and QRSC for the IBSC using the temperature characteristics of the *P*_OE_ value. By changing the host material from GaAs to AlGaAs, a high *P*_OE_ value was obtained. Among the samples in the present study, the GaSb/AlGaAs QDSC showed the best temperature characteristics with respect to the *P*_OE_ value. This result is attributed to the formation of a strong quantum confinement structure in the GaSb/AlGaAs QDSC, which suppresses the unintended escape of carriers in the IB as a result of thermal excitation.

## Figures and Tables

**Table 1:** Mean diameter, height, and density of GaSb QDs and QRs grown on an AlGaAs layer at 460 °C.

Structure	Diameter (nm)	Height (nm)	Density (cm^−2^)
QDs	27.84	2.56	7.7 × 10^10^
QRs	28.44	0.95	7.4 × 10^10^

**Table 2:** Parameters of the light current–voltage curves for the GaSb/AlGaAs QDSCs and QRSCs.

SC	J_SC_ (mA/cm^2^)	V_OC_ (V)	FF (-)
GaSb/AlGaAs QD	1.13	0.596	0.351
GaSb/AlGaAs QR	3.83	0.679	0.389
